# Quality evaluation of short videos about insomnia on TikTok and Bilibili: A cross-sectional study

**DOI:** 10.1097/MD.0000000000050806

**Published:** 2026-10-09

**Authors:** Yubo Yang, Minghao Zhou, Deyu Jiang, Roujia Sha, Zhiwei Huang, Jingan Tan, Yutao Hou

**Affiliations:** a School of Acupuncture and Moxibustion, Shandong University of Traditional Chinese Medicine, Jinan, Shandong Province, China; b School of Pharmacy, Shandong University of Traditional Chinese Medicine, Jinan, Shandong Province, China; c School of Chinese Medicine, Shandong University of Traditional Chinese Medicine, Jinan, Shandong Province, China; d School of Public Health, Shandong University of Traditional Chinese Medicine, Jinan, Shandong Province, China.

**Keywords:** health information dissemination, insomnia, short video platforms

## Abstract

This study systematically assesses the content quality and reliability of short videos related to insomnia on 2 major platforms, TikTok and Bilibili, and explores the influence of platform ecology and uploader identity on information dissemination. On November 22, 2025, searches were conducted on both platforms using the keyword “insomnia.” The top 100 relevant videos under the default ranking from each platform were included (final sample: 51 from Bilibili, 79 from TikTok). Two physicians independently assessed video quality using the Global Quality Scale, the modified DISCERN (mDISCERN) scale, and the Journal of the American Medical Association benchmark criteria. Video characteristics, interaction data, and user comment texts were analyzed. The overall quality of the included videos was moderate (median Global Quality Scale = 3.0; median mDISCERN = 3.5). Platform differences were significant: Bilibili videos were longer (median duration 975 seconds) and had higher mDISCERN and Journal of the American Medical Association scores than TikTok videos (all *P* < .001). TikTok videos received more likes (*P* < .001). Content from designated health professionals demonstrated the highest scientific quality (*P* < .001). User interaction metrics (comments, shares) showed no significant correlation with video scientific quality (prediction model *R*^2^ ≈ 0), indicating that popularity does not reflect reliability. The quality of insomnia-related short videos varies with platform ecology and uploader identity. Professional sources are key to ensuring scientific quality; however, public interaction data (likes, comments) primarily reflect emotional resonance rather than content quality. Differentiated platform governance, enhanced cross-platform narrative skills for creators, and strengthened public education on critical digital health literacy are recommended.

## 1. Introduction

Insomnia is a common sleep disorder primarily characterized by difficulty initiating sleep,^[1]^ difficulty maintaining sleep, or early morning awakening, accompanied by symptoms such as daytime fatigue, impaired attention, and emotional distress. Based on duration, insomnia is categorized as acute or chronic. Acute insomnia typically lasts for a shorter period (<3 months) and is often triggered by stressful events. Chronic insomnia (also known as insomnia disorder) refers to symptoms occurring at least 3 nights per week and persisting for at least 3 months, often accompanied by impaired daytime functioning.^[2]^ Both types clinically manifest mainly as sleep initiation or maintenance difficulties, frequently accompanied by daytime fatigue, attention decline, and emotional distress.^[3]^ The overall prevalence of insomnia among adults is high, approximately 33% to 50%,^[4–6]^ with a particularly significant impact on women, the elderly, and individuals with chronic diseases.^[7,8]^ Without standardized intervention, chronic insomnia not only severely reduces quality of life but also increases the risk of cardiovascular diseases, depression, and anxiety disorders. It may even lead to accidents due to daytime sleepiness, thereby imposing a heavy burden on individuals and society.^[9]^ Regarding treatment, international authoritative guidelines (such as those from the American Academy of Sleep Medicine) recommend cognitive behavioral therapy for insomnia (CBT-I) as the first-line treatment for chronic insomnia.^[10]^ Therefore, disseminating effective, reliable, and evidence-based health information to the public – particularly information that accurately distinguishes insomnia types and promotes standardized treatments such as CBT-I – is crucial for disease prevention, early identification, and improving treatment adherence. This has become a key public health issue.

In recent years, the way the public accesses health information has undergone a significant shift, with short video platforms such as TikTok and Bilibili becoming primary channels. Notably, these 2 platforms differ fundamentally in positioning, user base, and content ecology. TikTok is an entertainment-oriented, algorithm-driven short video platform that prioritizes the breadth and efficiency of information dissemination, whereas Bilibili is known for its high-engagement community culture and encouragement of in-depth content. These differences may lead to distinctly different presentations of health information. Although they provide new opportunities for medical science communication, content production on such platforms often lacks strict academic review processes, potentially leading to uneven information quality and increasing the risk of disseminating erroneous or one-sided information.^[11]^ Studies on other health topics, such as pancreatic cancer and pediatric pneumonia, indicate that while such content often has high interaction metrics, it may lack scientific rigor and educational value, and could even exacerbate audience anxiety or lead to inappropriate self-management.^[12–14]^ Given the significant health impact of insomnia and the professional requirements for its diagnosis and treatment (e.g., distinguishing between acute/chronic types and the priority of CBT-I),^[15]^ combined with the potential misinformation risks on short video platforms, systematically evaluating the content quality and reliability of insomnia-related videos on these platforms is particularly urgent. This study focuses on TikTok and Bilibili, aiming to assess the quality and reliability of their insomnia-related content.

We pay special attention to whether video content accurately distinguishes between different types of insomnia and whether its treatment recommendations align with evidence-based medical principles centered on CBT-I. By elucidating the scientific nature, reliability, and potential risks of these videos, this study aims to provide empirical evidence for future standardization of online health information dissemination and improvement of public health literacy.

## 2. Methods

### 2.1. Medical ethics

This study is an observational analysis based on publicly available, nonsensitive data and does not constitute human subject research requiring Institutional Review Board approval. All data were collected from publicly accessible short videos and their associated comments on the TikTok and Bilibili platforms. Researchers did not interact with any users, did not intervene in the platform environment, and did not obtain any private information.

To protect user privacy, all collected comments underwent strict deidentification and anonymization before analysis. The procedure included removal of all usernames, user accounts, personal profile links, and other identifying metadata; screening and replacement of potentially existing personal information (e.g., phone numbers, locations) using regular expressions; and aggregation of all data, with analysis conducted only at the group level, eliminating any possibility of tracing data back to specific users. This study focuses solely on the content and emotional tendencies of comment texts, not on individual commenter characteristics. The specific content is provided in Supplemental File 1, Supplemental Digital Content 1.

This study adheres to the terms of service of each platform and relevant data protection regulations. The study design and ethical considerations are consistent with established norms for content analysis using publicly available social media data.

Furthermore, the methodology complies with the Internet Research Ethics Framework proposed by the Association of Internet Researchers, which states that analyzing publicly archived anonymous data with no researcher-user interaction constitutes low-risk research and generally does not require formal Institutional Review Board review.

### 2.2. Video acquisition

This study aims to evaluate the quality of video content encountered when users proactively search for insomnia-related information in the most direct and common way. “Insomnia” is the most standard and widely recognized medical and public term for this condition in the Chinese context. Using this single keyword for retrieval simulates the real scenario of an ordinary user conducting an initial search without professional knowledge, ensuring the representativeness of the sample for simulating general public information exposure. Using a single keyword also ensures that the initial video pools from both platforms are retrieved under identical conditions, providing a comparable basis for cross-platform analysis and avoiding the complexity introduced by multiple keyword combinations.

Therefore, on November 22, 2025, researchers used the keyword “insomnia” to search Bilibili and TikTok. To minimize the impact of personalized recommendations, searches were performed using newly registered accounts with no search history. The study included the top 100 videos related to insomnia under each platform’s default sorting. According to relevant research, videos ranked beyond 100 on a single platform have no significant impact on analysis results.^[16–18]^ Videos were included if they directly addressed the etiology, symptoms, diagnosis, treatment, or prevention of insomnia and were publicly accessible (excluding private or restricted content). Videos identified as promotional material, clickbait, or unrelated to insomnia were excluded. Finally, 51 Bilibili videos and 79 TikTok videos were retained, totaling 130 videos included in the study (Fig. 1).

**Figure 1.**
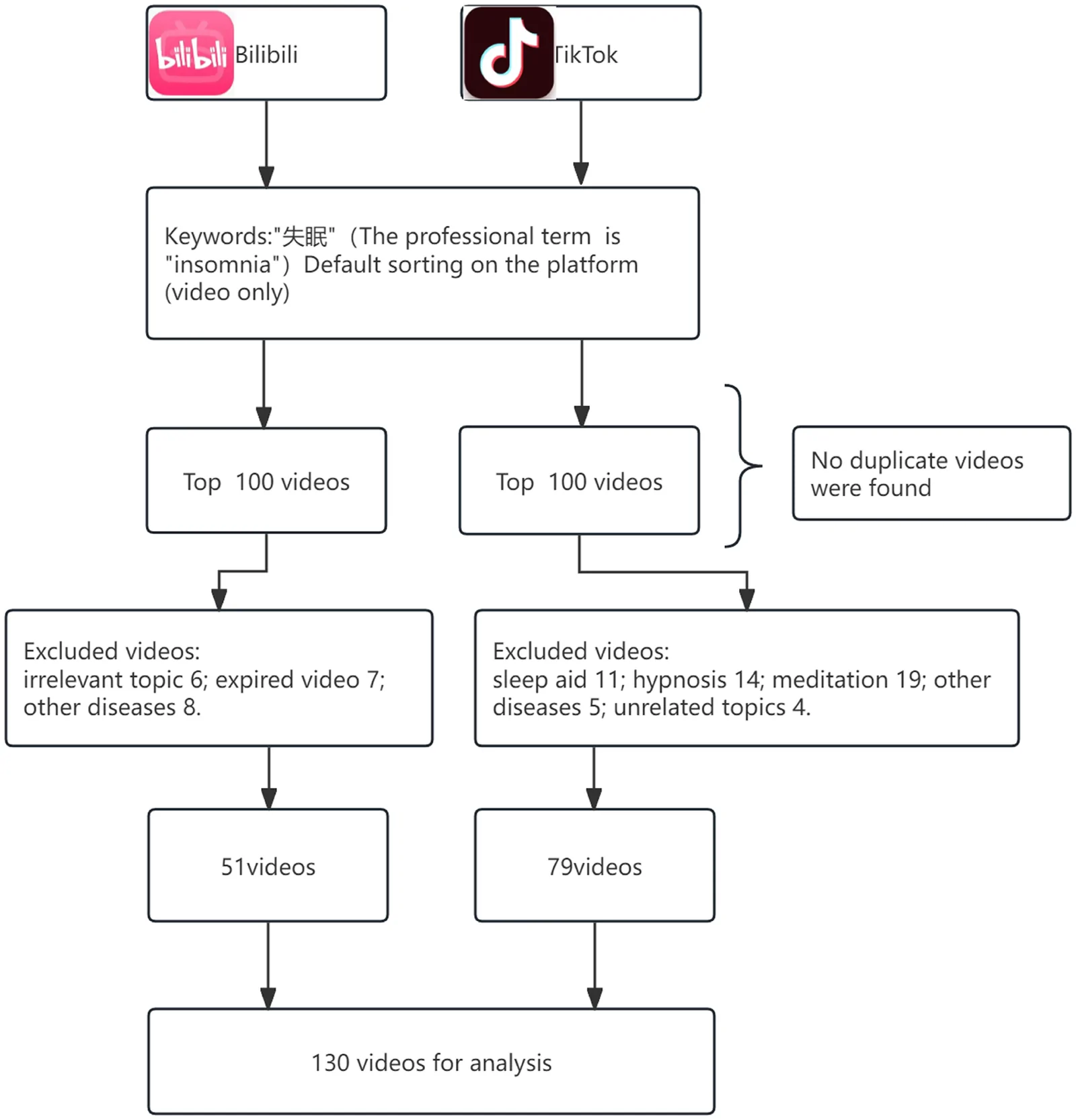
Inclusion and exclusion process for short videos.

### 2.3. Video baseline information data collection and classification

This study collected general information, content, and quality data for the included insomnia-related videos on Bilibili and TikTok. Data covered video duration, likes, favorites, comments, shares, content aspects (etiology, clinical manifestations, diagnosis, treatment, prognosis), and quality indicators (Global Quality Scale [GQS], modified DISCERN [mDISCERN], Journal of the American Medical Association [JAMA]). Video uploaders were classified into 3 categories: non-designated health professionals (NDHPs), designated health professionals (DHPs), and individual users. The aforementioned indicators were analyzed for each category.

### 2.4. Comment text collection and preprocessing

All original user comment texts from the 130 target videos were collected as of November 5, 2025, with an initial sample size of N = 4205. To meet the needs of subsequent multidimensional analysis, systematic cleaning and preprocessing were performed on the raw data. The workflow was as follows: first, unified data normalization was performed, including converting all text to lowercase and removing URLs, emojis, special characters, and irrelevant numbers. Subsequently, targeted processing was conducted according to different analysis purposes.

For Word Cloud and Semantic Network Graph Analysis, the aim was to extract core thematic words to visualize the focus of public discussion. The cleaning process included using a precise Chinese word segmentation tool (e.g., Jieba) for tokenization, constructing and applying a customized stopword list (covering general stopwords, modal particles, auxiliaries, platform-specific language, and high-frequency but semantically empty internet slang), and ultimately retaining content words such as nouns and verbs.

For modeling analysis of comment features and video quality scores (JAMA, GQS, mDISCERN), the aim was to construct features for quantitative models. After basic tokenization and stopword removal, lemmatization was performed to unify word forms, and the text was converted into structured numerical features. This study employed the Bag-of-Words model combined with TF-IDF weighting, or pretrained word vector representations, to convert each comment into a feature vector for input into subsequent regression or classification models.

All cleaning steps were automated through a pipeline implemented in the Python programming environment (utilizing libraries such as pandas, jieba, and scikit-learn), ensuring process reproducibility and result consistency, thus providing high-quality, structured text data for subsequent visualization and statistical modeling.

### 2.5. Video quality assessment

Two experts (both psychiatry resident physicians who have completed standardized training and hold practicing physician certificates) independently assessed the quality of the included videos. The JAMA benchmark criteria,^[19]^ the GQS,^[20]^ and the mDISCERN scale^[21]^ were used to evaluate video quality and reliability. Subjective scoring was based on international guidelines related to insomnia. If the scores from the 2 experts were inconsistent, a third arbitrator provided the final score; if consistent, the average of the 2 scores was taken. The relevant scale tools are as follows:

The GQS is a 5-point Likert scale (1 = poor quality, 5 = excellent quality) used to assess the overall quality, transparency, and usability of information (Table 1).
The mDISCERN scale was adapted for video content and includes 5 items assessing reliability. Each item is scored dichotomously (yes = 1, no = 0), with a total score range of 0 to 5 (Table 2).
The JAMA benchmark assesses information quality based on 4 core principles: authorship, attribution, currency, and disclosure. Each principle is scored dichotomously (yes = 1, no = 0), with a total score range of 0 to 4 (Table 3).

**Table 1:** The Global Quality Score (GQS) quality criteria.

Item features	Points
Poor quality; poor flow of the videos; most information missing; not at all useful for patients	1
Generally poor quality; some information listed, but many important topics missing; of very limited use to patients	2
Moderate quality; suboptimal flow; some important adequately discussed, but other information poorly discussed; somewhat useful for patients	3
Good quality and generally good flow; most of the relevant information listed, but some topics not covered; useful for patients	4
Excellent quality and flow; very useful for patients	5

**Table 2:** The modified DISCERN (mDISCERN) quality criteria.

Reliability score
1. Is the video clear, concise, and understandable?
2. Are valid sources cited?
3. Is the content presented balanced and unbiased?
4. Are additional sources of content listed for patient reference?
5. Are areas of uncertainty mentioned?

**Table 3:** The Journal of the American Medical Association (JAMA) benchmark criteria.

Score	Score component	
1 score	Authorship	Author and contributor credentials and their affiliations should be provided.
1 score	Attribution	Clearly lists all copyright information and states references and sources for content.
1 score	Currency	Initial date of posted content and subsequent updates to content should be provided.
1 score	Disclosure	Conflicts of interest, funding, sponsorship, advertising, support, and video ownership should be fully disclosed.

### 2.6. Statistical analysis

The Shapiro–Wilk test was used to test the normality of continuous variables. Normally distributed continuous variables are expressed as mean ± standard deviation, while non-normally distributed continuous variables are expressed as median and interquartile range (IQR; M [P25, P75]). Categorical variables are expressed as numbers and proportions. Cohen’s κ was used to quantify inter-rater agreement. Comparisons of non-normally distributed continuous variables were performed using the Mann–Whitney *U* test and the Kruskal–Wallis test. Spearman correlation analysis was used to assess the relationship between audience interaction and video quality. Statistical analysis and figure generation were performed using Python version 3.13.5 (Python Software Foundation, Beaverton).

## 3. Results

### 3.1. Inter-rater reliability analysis

The inter-rater reliability calculation resulted in Cohen’s kappa (κ) ≈ 0.78, indicating good agreement between the 2 expert raters.

### 3.2. Video baseline characteristics

A total of 130 insomnia-related short videos were included for analysis. The median video duration was 501 seconds (IQR: 319–1197). Median user interaction data were as follows: likes 80.5 (IQR: 28–500), favorites 476.5 (IQR: 50–1500), comments 939.5 (IQR: 200–5000), and shares 354 (IQR: 100–500). Regarding video content, the most covered topic was treatment (n = 32, 24.6%), followed by clinical manifestations (n = 27, 20.8%), etiology (n = 26, 20.0%), prognosis (n = 24, 18.5%), and diagnosis (n = 21, 16.2%). The median overall quality scores were GQS 3.0 (IQR: 3.5–4.5), mDISCERN 3.5 (IQR: 3.0–4.0), and JAMA 3.0 (IQR: 3.0–4.0; Table 4).

**Table 4:** General characteristics, quality, and reliability of the insomnia videos.

Variables	Total (n = 130)
General information	
Video length (s), M (Q1, Q3)	501 (319, 1197)
Likes, M (Q1, Q3)	80.5 (28, 500)
Collections, M (Q1, Q3)	476.5 (50, 1500)
Comments, M (Q1, Q3)	939.5 (200, 5000)
Shares, M (Q1, Q3)	354 (100, 500)
Video content	
Etiology	26
Clinical manifestation	27
Diagnosis	21
Treatment	32
Prognosis	24
Video quality	
GQS score, M (Q1, Q3)	3.0 (3.5, 4.5)
mDISCERN score, M (Q1, Q3)	3.5 (3.0, 4.0)
JAMA score, M (Q1, Q3)	3.0 (3.0, 4.0)

All the *P*-values were obtained from the Mann–Whitney *U* test.

GQS = Global Quality Scale, JAMA = Journal of the American Medical Association, mDISCERN = modified DISCERN.

### 3.3. Comparison of video characteristics and quality between platforms

Bilibili and TikTok showed significant differences in basic video features, content coverage, and quality scores. The median video duration on Bilibili was significantly higher than on TikTok (975 seconds vs 81 seconds, *P* < .001). In terms of interaction data, the median number of likes for TikTok videos (9853) was significantly higher than for Bilibili videos (1444, *P* < .001), while the number of comments (108 vs 62) and shares (586 vs 407) on Bilibili were significantly higher than on TikTok (both *P* < .001). Regarding content coverage, both platforms were dominated by the treatment theme (Bilibili: 14; TikTok: 19), followed by prognosis and clinical manifestations. In terms of quality scores, the median mDISCERN score for Bilibili videos was significantly higher than for TikTok videos (4.0 vs 3.0, *P* < .001), and the JAMA score was also higher (*P* < .001). TikTok’s GQS score was slightly higher than Bilibili’s (3.0 vs 3.5, *P* = .002; Table 5).

**Table 5:** General information, quality, and reliability scores of insomnia videos on TikTok and Bilibili.

Variables	Bilibili (n = 51)	TikTok (n = 79)	*P*-value
General information			
Video length (s), M (Q1, Q3)	975 (285, 1280)	81 (34, 100)	**<.001**
Likes, M (Q1, Q3)	1444 (614, 11,946)	9853 (288, 12,000)	**<.001**
Collections, M (Q1, Q3)	1557 (775, 9933)	5476 (87, 24,300)	**<.001**
Comments, M (Q1, Q3)	108 (79, 662)	62 (8, 262)	**<.001**
Shares, M (Q1, Q3)	586 (126, 2279)	407 (27, 1078)	**<.001**
Video content			
Etiology	10	16	–
Clinical manifestation	10	15	–
Diagnosis	10	14	–
Treatment	14	19	–
Prognosis	17	15	–
Video quality			
GQS score, M (Q1, Q3)	3.5 (3.0, 4.0)	3.0 (3.5, 4.5)	.002
mDISCERN score, M (Q1, Q3)	4.0 (4.0, 4.5)	3.0 (3.0, 4.0)	**<.001**
JAMA score, M (Q1, Q3)	3.0 (2.0, 3.5)	3.0 (3.0, 4.0)	**<.001**

Bold *P*-values represent significant differences between Bilibili and TikTok.

All the *P*-values were obtained from the Mann–Whitney *U* test and significance was set at *P* < .05.

GQS = Global Quality Scale, JAMA = Journal of the American Medical Association, mDISCERN = modified DISCERN.

### 3.4. Video uploader characteristics

Uploaders were classified by institution/individual status, professional certification status, and category (NDHPs, DHPs, or individual users). On TikTok, uploaders were predominantly individual accounts (Fig. 2A), with professionally certified accounts comprising 82% (Fig. 2B). Among these, 37 were doctor accounts, while patient uploaders and other types were relatively few (Fig. 2C). On Bilibili, institutional accounts comprised one-third of uploaders (Fig. 2A), and accounts without medical certification accounted for 93% (Fig. 2B). The numbers of doctor uploaders, patient group uploaders, and other uploaders were relatively balanced (Fig. 2C). Among both certified and noncertified uploaders, the male-to-female ratio was relatively balanced, and institutions were the main publishing entities (Fig. 2D).

**Figure 2.**
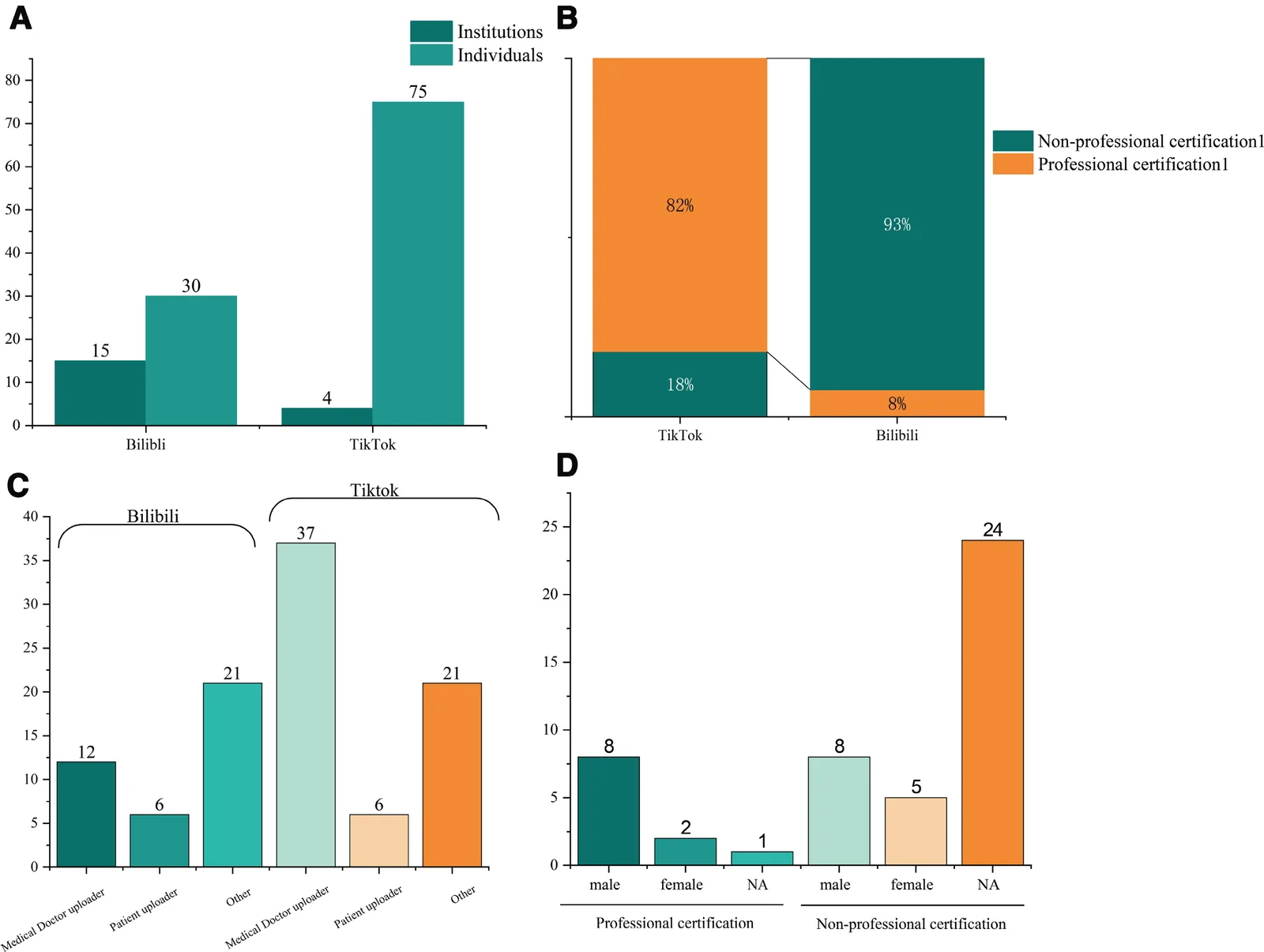
Relevant information of the video uploader. (A) Video sources: institutions versus individuals; (B) professional certification proportions; (C) gender distribution of uploaders; (D) gender and certification cross-tabulation.

In the analysis comparing NDHPs, DHPs, and individual users, NDHPs had significantly higher numbers of likes, favorites, and comments compared to DHPs and individual users (all *P* < .001). Both NDHPs and DHPs had significantly higher share counts than individual users (*P* < .001). Regarding video quality, the GQS and mDISCERN scores of DHPs were significantly higher than those of NDHPs and individual users (*P* < .001). For JAMA scores, differences among the 3 groups were small and not statistically significant (Table 6).

**Table 6:** Characteristics, quality, and reliability of insomnia videos by different uploaders on TikTok and Bilibili.

Variables	NDHPs (n = 36)	DHPs (n = 36)	Individual users (n = 35)	*P*_N-D_	*P*_N-I_	*P*_D-I_
Video length (s), M (Q1, Q3)	354 (100, 500)	354 (100, 500)	354 (100, 500)	.023	**<.001**	.008
Likes, M (Q1, Q3)	1492 (327, 12,573)	1295 (319.5, 11,973)	1295 (310, 10,023)	.045	**<.001**	.032
Collections, M (Q1, Q3)	939 (159, 5211)	690 (213, 5034)	934 (634, 5000)	.067	**<.001**	.025
Comments, M (Q1, Q3)	80 (28, 500)	327 (17, 620)	805 (57, 890)	.089	**<.001**	.041
Shares, M (Q1, Q3)	476 (50, 1500)	437 (50, 1500)	3664 (10, 4000)			
GQS score, M (Q1, Q3)	3.5 (3.0, 4.5)	4.0 (3.5, 4.5)	3.0 (3.0, 4.5)	**<.001**	.023	.005
mDISCERN score, M (Q1, Q3)	3.0 (3.0, 4.0)	3.5 (3.0, 4.0)	3.5 (3.0, 4.0)	**<.001**	.856	**<.001**
JAMA score, M (Q1, Q3)	3.0 (3.0, 4.0)	3.0 (3.0, 4.0)	3.0 (3.0, 4.0)	.008	.934	.007

Bold *P*-values represent significant differences among the 3 uploader groups.

All the *P*-values were obtained from the Mann–Whitney *U* test and significance was set at *P* < .05.

DHPs = designated health professionals, GQS = Global Quality Scale, JAMA = Journal of the American Medical Association, mDISCERN = modified DISCERN, NDHPs = non-designated health professionals.

### 3.5. Video content characteristics

Video content was categorized into 6 types: etiology, symptoms, diagnosis, treatment, prevention, and epidemiological information. On TikTok, only 25.3% of videos covered these 6 content types, with treatment being the most common (15.2%) and epidemiological information lacking (Fig. 3A). On Bilibili, 48.1% of videos covered all 6 categories, with treatment being the most common at 28.8% (Fig. 3B).

**Figure 3.**
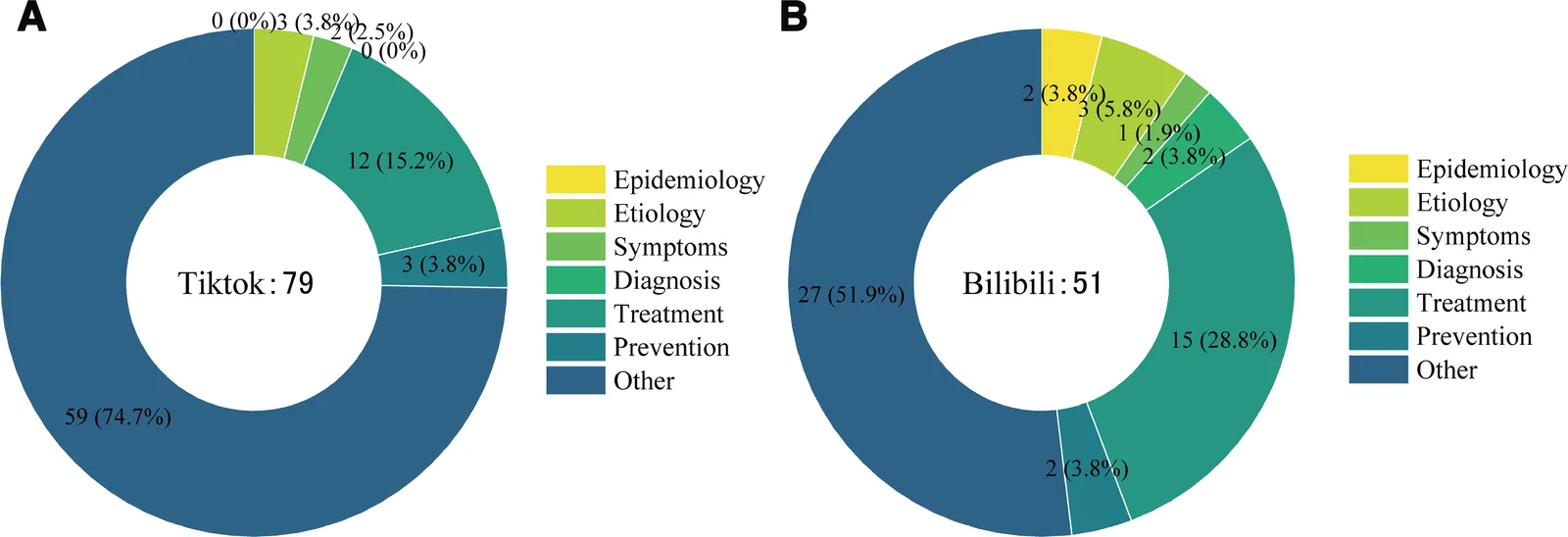
Comparison of the classification distribution of health video content on TikTok and Bilibili. (A) Proportion of content categories on TikTok; (B) proportion of content categories on Bilibili.

### 3.6. Video uploader and platform difference analysis

Violin plots were used to assess the distribution of GQS, mDISCERN, and JAMA scores among uploaders categorized as medical institutions or individuals, and further subdivided into individual users, NDHPs, and DHPs. When comparing institutional and individual medical uploaders, the median and IQR for GQS and mDISCERN scores were identical (GQS: 3.0 [IQR: 2.5–3.5]; mDISCERN: 4.0 [IQR: 3.0–4.5]), with no statistically significant differences (Fig. 4A and B). For JAMA scores, the distribution for institutional uploaders was more concentrated, while that for individual medical uploaders was more dispersed with more outliers; the difference between groups was statistically significant (*P* < .0001; Fig. 4C).

**Figure 4.**
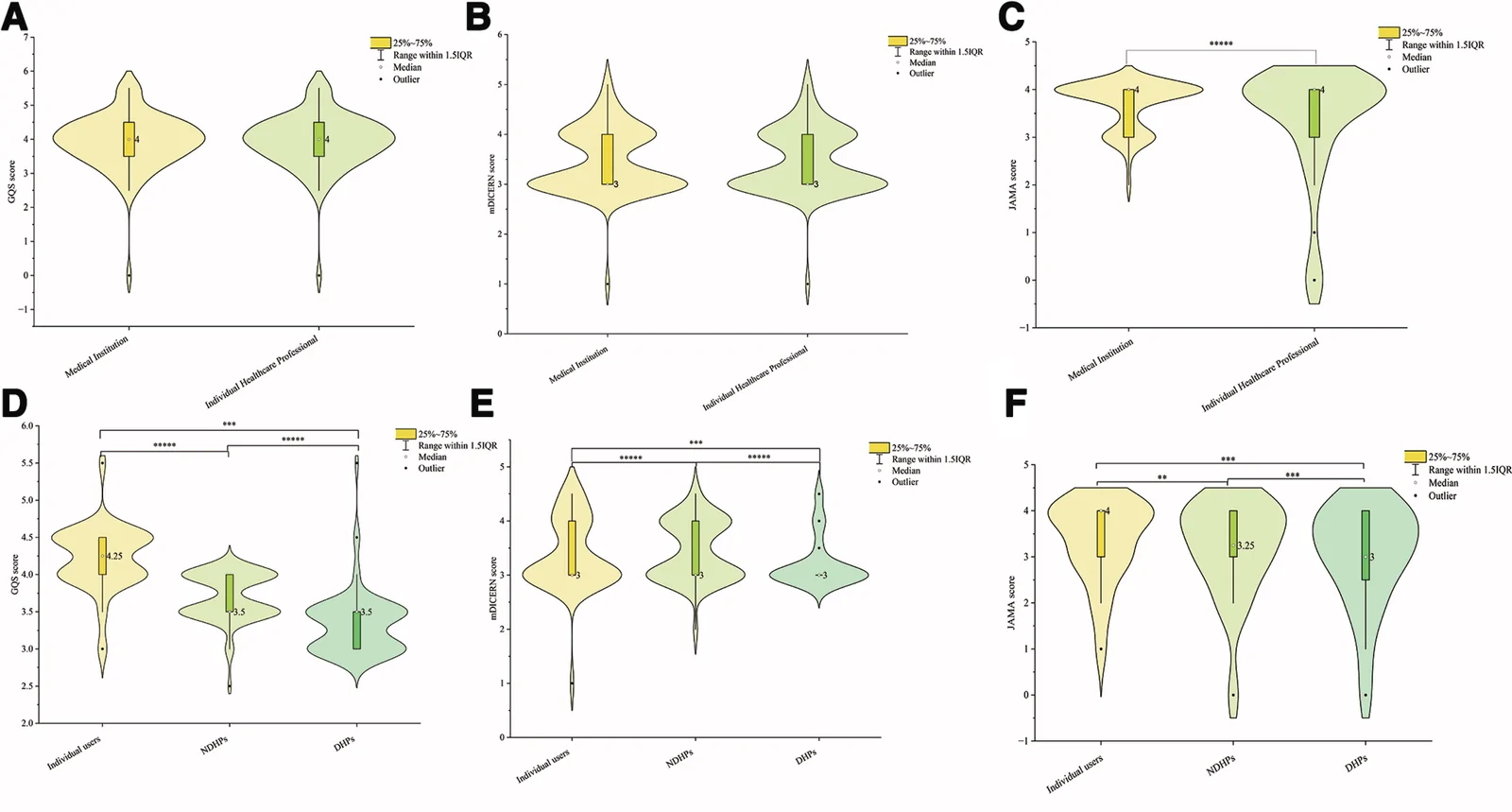
Scoring conditions for different platforms and different uploader identities. (A) Distribution of GQS scores: medical institutions versus individual medical uploaders; (B) distribution of mDISCERN scores: medical institutions versus individual medical uploaders; (C) distribution of JAMA scores: medical institutions versus individual medical uploaders; (D) distribution of GQS scores: individual users, NDHPs, and DHPs; (E) distribution of mDISCERN scores: individual users, NDHPs, and DHPs; (F) distribution of JAMA scores: individual users, NDHPs, and DHPs. DHPs = designated health professionals, GQS = Global Quality Scale, IQR = interquartile range, JAMA = Journal of the American Medical Association, mDISCERN = modified DISCERN, NDHPs = non-designated health professionals.

In comparisons among individual users, NDHPs, and DHPs, statistically significant differences (*P* < .01) were found across all score types. Individual users had the highest median GQS score (4.25 [IQR: 3.0–4.5]; Fig. 4D). For mDISCERN scores, DHPs showed a notably concentrated distribution without a clear IQR, differing from the broader distributions of the other 2 categories (Fig. 4E). For JAMA scores, individual users showed a relatively high median of 4 (IQR: 3.0–4.0), with total scores ranging from 3 to 4 across all 3 categories (Fig. 4F).

In addition, we analyzed the types of misinformation present in the videos. The most common misinformation involved unproven remedies (e.g., herbal supplements without evidence), followed by oversimplified causal explanations and exaggerated treatment claims. The distribution of misinformation types across platforms is shown in Figure 5.

**Figure 5.**
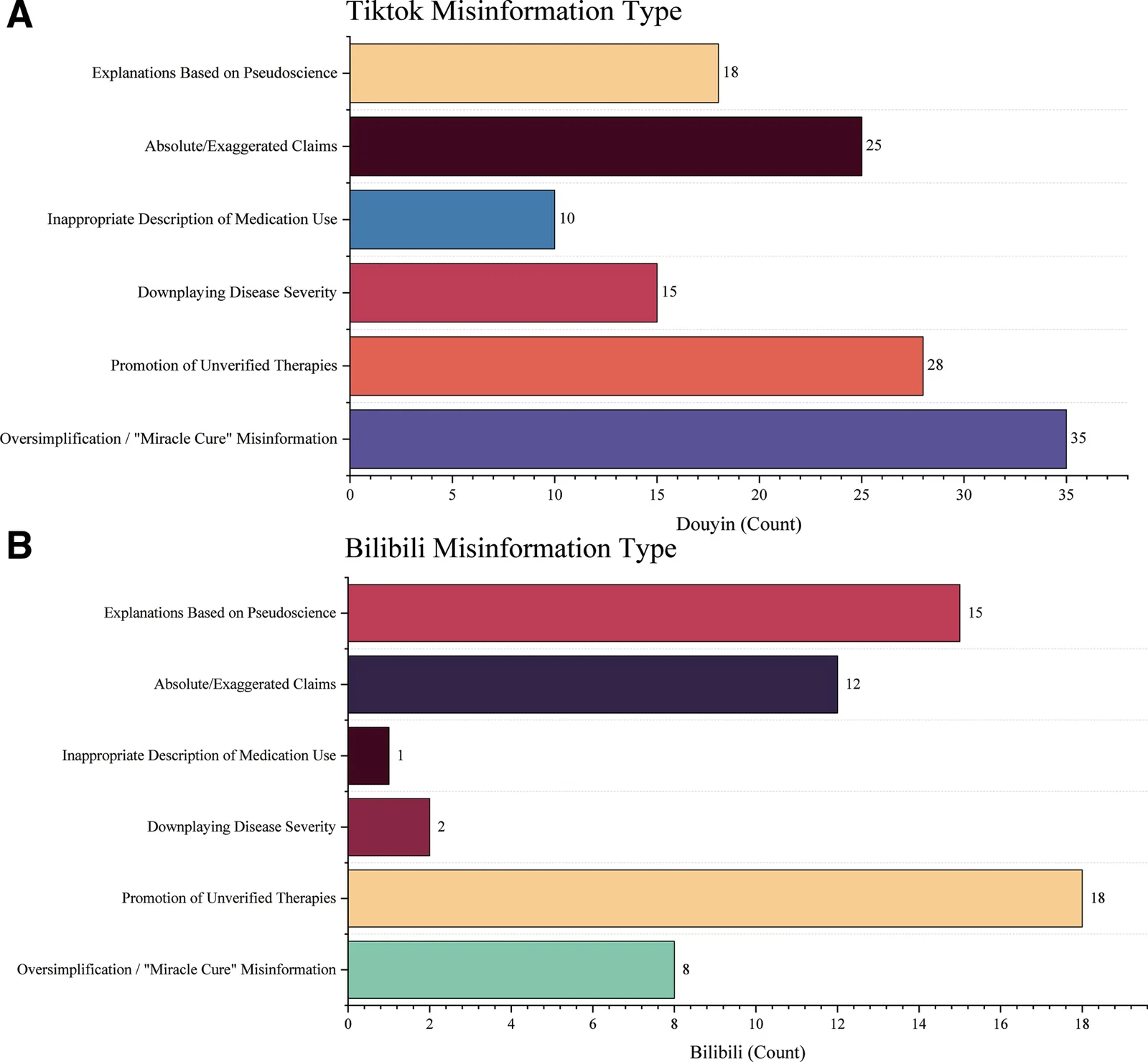
The misinformation type of the TikTok/Bilibili video content. (A) Distribution of TikTok misinformation types by percentage; (B) distribution of Douyin misinformation types by percentage.

### 3.7. Correlation analysis

For Bilibili, video duration was positively correlated with GQS score (*R* = 0.43, *P* < .001). The number of shares was negatively correlated with mDISCERN score (*R* = −0.32, *P* = .02) and not correlated with JAMA score. For TikTok, the number of shares was positively correlated with JAMA score (*R* = 0.29, *P* = .02; Table 7).

**Table 7:** The correlation between user interaction data on TikTok and Bilibili and ratings on GQS, mDISCERN, and JAMA scales.

Spearman *R*, *P*-value	Bilibili (n = 51)			TikTok (n = 79)		
	GQS	JAMA	mDISCERN	GQS	JAMA	mDISCERN
Likes	−0.13 (*P* = .34)	−0.08 (*P* = .56)	−0.31 (*P* = .02)	−0.09 (*P* = .47)	−0.08 (*P* = .53)	−0.09 (*P* = .47)
Collections	−0.11 (*P* = .43)	−0.08 (*P* = .57)	−0.28 (*P* = .04)	−0.11 (*P* = .37)	−0.09 (*P* = .48)	−0.09 (*P* = .48)
Comments	−0.11 (*P* = .43)	−0.07 (*P* = .61)	−0.29 (*P* = .03)	−0.12 (*P* = .34)	−0.1 (*P* = .44)	−0.09 (*P* = .48)
Shares	−0.13 (*P* = .35)	−0.08 (*P* = .56)	−0.32 (*P* = .02)	0.05 (*P* = .68)	0.29 (*P* = .02)	0.18 (*P* = .15)
Video length (s)	0.43 (*P* < .001)	0.19 (*P* = .17)	0.07 (*P* = .62)	−0.08 (*P* = .53)	−0.12 (*P* = .33)	−0.06 (*P* = .63)

All the *P*-values were obtained from the Spearman test.

GQS = Global Quality Scale, JAMA = Journal of the American Medical Association, mDISCERN = modified DISCERN.

### 3.8. Keyword features of insomnia-related comments on Bilibili and TikTok platforms

In the keyword co-occurrence network for Bilibili comments, core high-frequency keywords included “sleep” (n = 43), “night” (n = 33), “can’t sleep” (n = 33), “listen” (n = 31), and “like” (n = 28). Word frequencies were tiered as very high (≥33), high (25–33), medium (16–25), and low (8–16). “Sleep” and “night” belonged to the very high tier (Fig. 6A). High-frequency keywords showed a concentrated distribution and tight interconnections. In the core word cloud (Fig. 6B), high-frequency words included “insomnia,” “light,” and “Suan Zao Ren Tang.” Keywords were categorized into themes such as sleep state (e.g., “light sleep” and “wake up”), intervention methods (e.g., “meditation” and “guided breathing”), symptoms and emotions (e.g., “pressure” and “anxiety”), and related concepts (e.g., “circadian rhythm” and “Traditional Chinese Medicine”).

**Figure 6.**
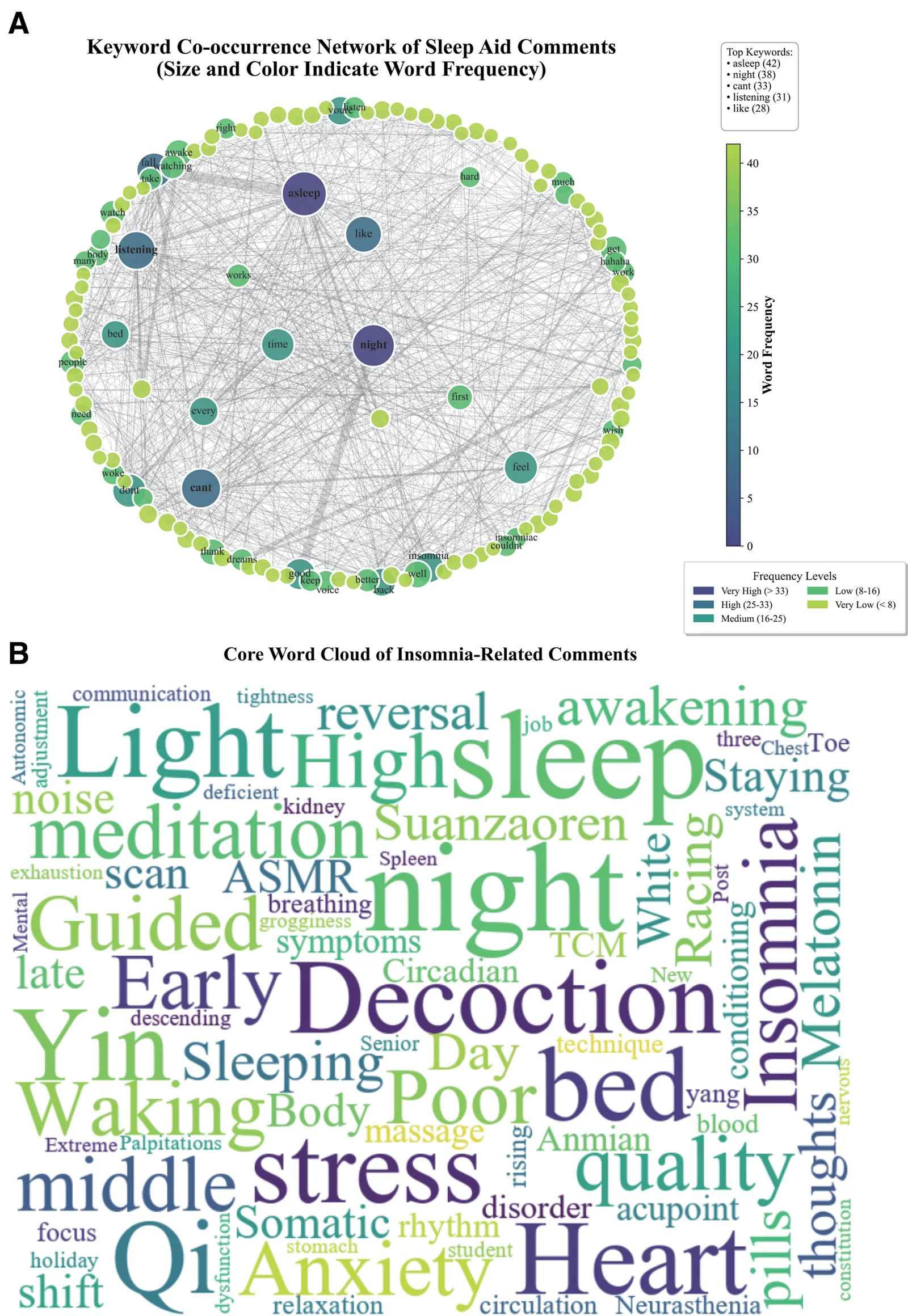
Analysis of Bilibili sleep aid and insomnia-related comments: keyword co-occurrence network and core word cloud. (A) Keyword co-occurrence network of sleep aid comments; (B) core word cloud of insomnia-related comments.

In the keyword co-occurrence network for TikTok comments (Fig. 7A), core keywords included “sleep” (frequency = 101), “fall” (91), “night” (82), “cant” (74), and “bed” (70). Frequencies were tiered as very high (≥80), high (60–80), medium (40–60), low (20–40), and very low (<20). “Sleep,” “fall,” and “night” belonged to the very high tier. High-frequency keywords showed a concentrated distribution and high connection density. In the core word cloud (Fig. 7B), high-frequency words included “sleep,” “night,” “Insomnia,” “breathing,” and “exhaustion.” Keywords were classified into themes such as sleep state (e.g., “asleep,” “sleeping,” and “waking”), intervention methods (e.g., “Meditation,” “Melatonin,” and “Zopiclone”), symptoms and emotions (e.g., “Anxiety,” “stress,” and “exhaustion”), and associated concepts (e.g., “Circadian” and “Traditional”).

**Figure 7.**
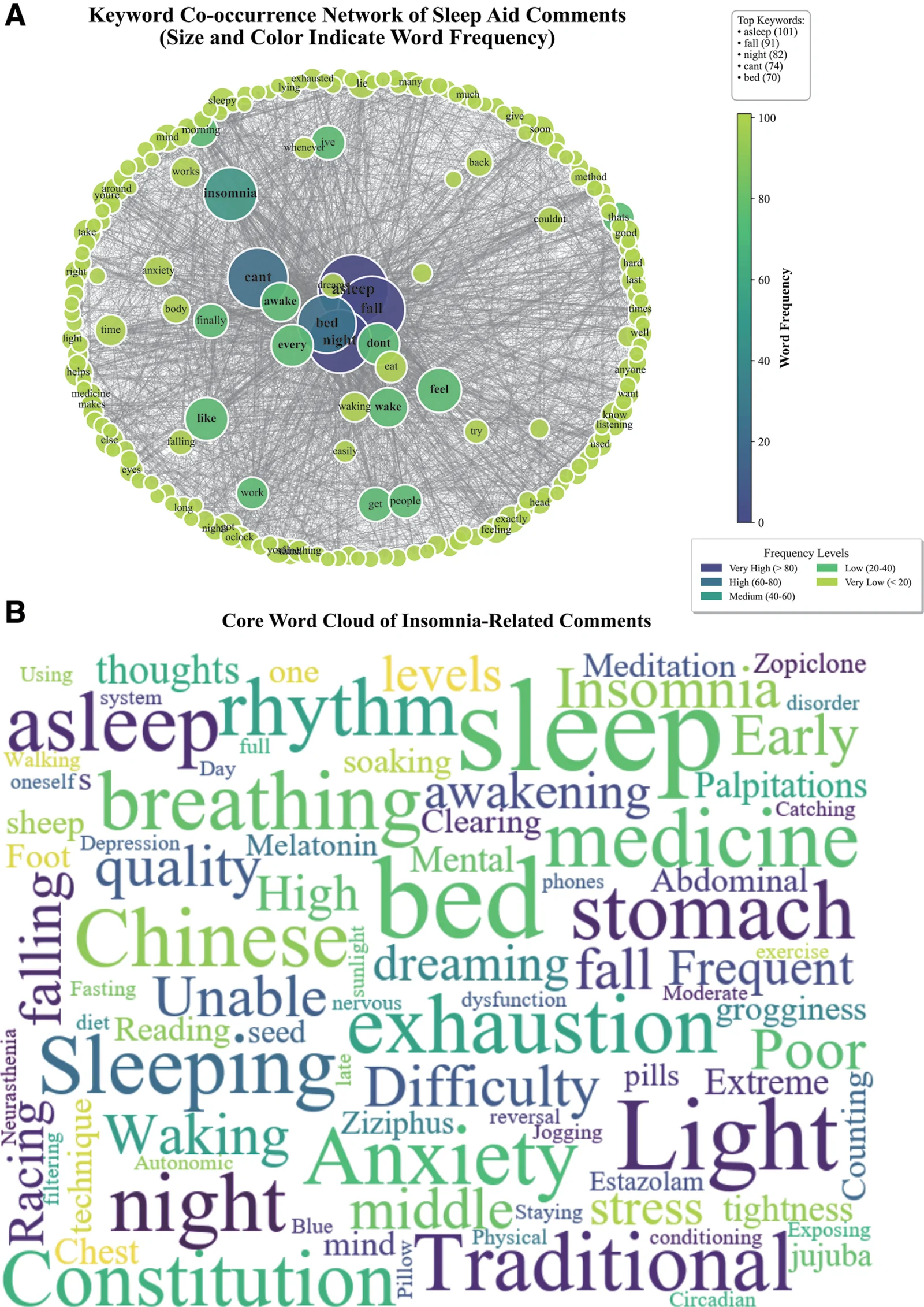
Analyzing TikTok sleep aid comments: keyword co-occurrence network and core word cloud. (A) Keyword co-occurrence network of sleep aid comments; (B) core word cloud of insomnia-related comments.

### 3.9. Analysis of prediction performance of video quality assessment models based on Bilibili and TikTok comments

For Bilibili comments, model training dynamics and prediction accuracy for JAMA, GQS, and mDISCERN scores were analyzed. Loss curves (Fig. 8A–C) showed decreasing trends for both training and validation loss across all 3 models as epochs increased. Mean absolute error (MAE) curves (Fig. 8D–F) also showed decreasing trends. However, the fit between predicted and actual values was poor: for JAMA scores, correlation was 0.092 (*R*^2^ = 0.0008); for GQS, correlation was 0.034 (*R*^2^ = −0.164); for mDISCERN, correlation was −0.099 (*R*^2^ = −0.173). Predicted values did not effectively fit actual values for any indicator (Fig. 8G–I).

**Figure 8.**
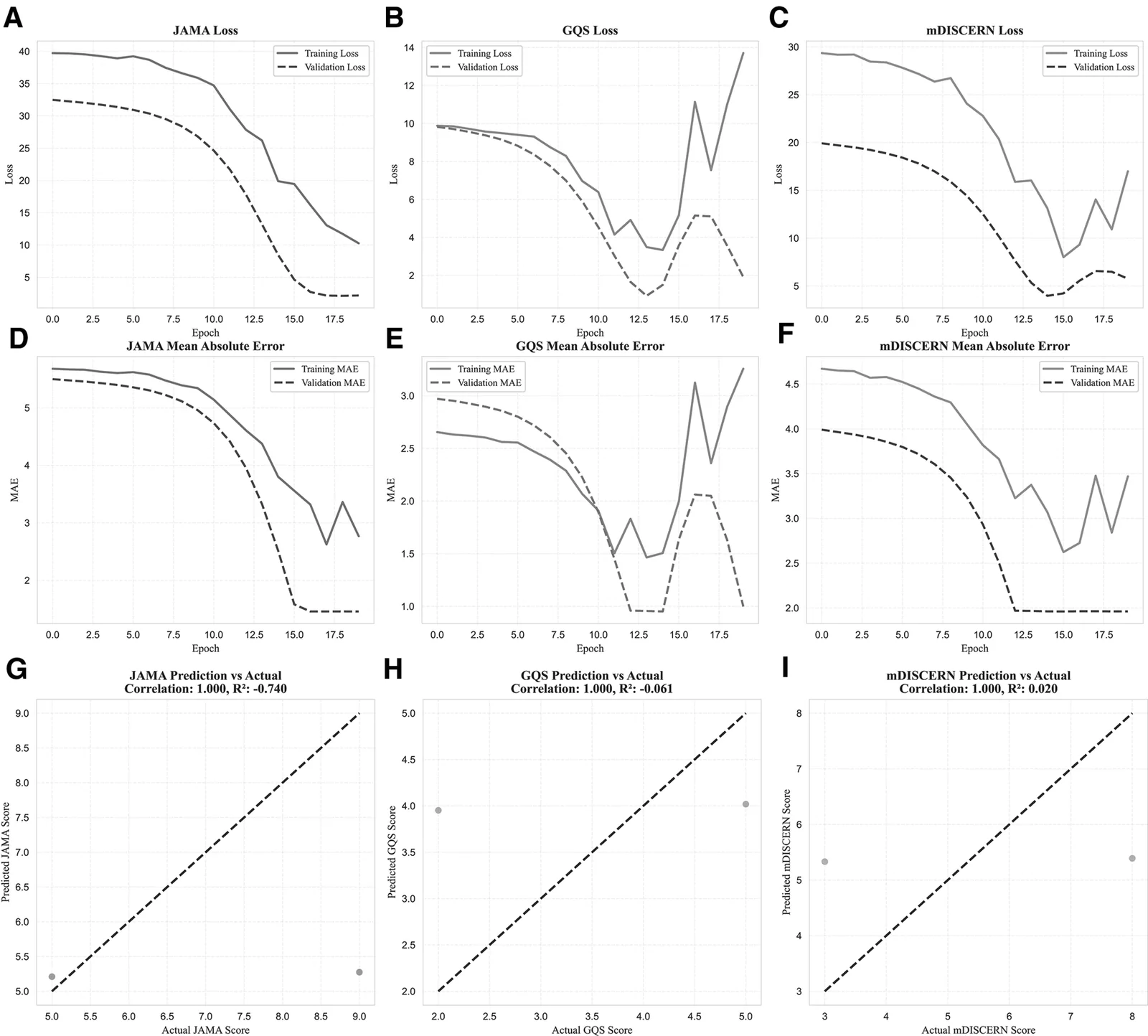
Model performance metrics and prediction accuracy across JAMA, GQS, and mDISCERN datasets from Bilibili. (A) Training and validation loss curves over epochs; (B) training and validation accuracy curves over epochs; (C) learning rate schedule over epochs; (D) training and validation MACE curves over epochs; (E) training and validation MAE curves over epochs; (F) training and validation MSE curves over epochs; (G) training and validation precision curves over epochs; (H) training and validation recall curves over epochs; (I) training and validation F1-score curves over epochs. GQS = Global Quality Scale, JAMA = Journal of the American Medical Association, MACE = major adverse cardiovascular events, MAE = mean absolute error, mDISCERN = modified DISCERN, MSE = mean squared error.

For TikTok comments, similar analyses were conducted. Loss curves (Fig. 9A–C) and MAE curves (Fig. 9D–F) showed decreasing trends. The fit between predicted and actual values was equally poor, with identical correlation and *R*^2^ values as the Bilibili models. In summary, although training loss and MAE decreased, the models’ predictions showed extremely poor fit with actual quality scores, resulting in low prediction accuracy for both platforms (Fig. 9G–I).

**Figure 9.**
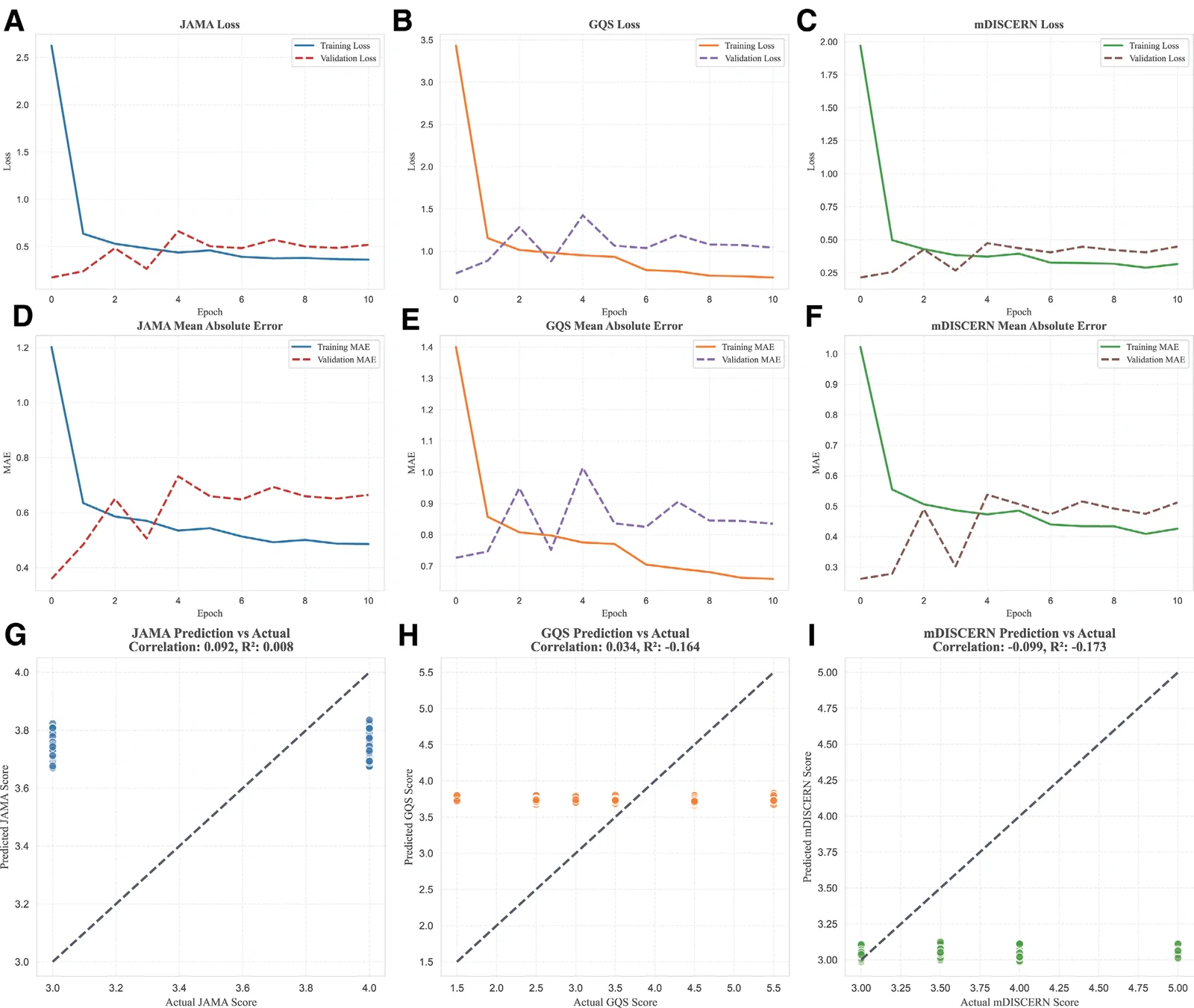
Model training dynamics and prediction accuracy for quality assessment metrics (JAMA, GQS, and mDISCERN). (A) Loss curves for JAMA score model (training and validation sets) over epochs; (B) loss curves for GQS score model (training and validation sets) over epochs; (C) loss curves for mDISCERN score model (training and validation sets) over epochs; (D) MAE curves for JAMA score model (training and validation sets) over epochs; (E) MAE curves for GQS score model (training and validation sets) over epochs; (F) MAE curves for mDISCERN score model (training and validation sets) over epochs; (G) correlation between predicted and actual JAMA scores (TikTok) over epochs; (H) correlation between predicted and actual GQS scores (TikTok) over epochs; (I) correlation between predicted and actual mDISCERN scores (TikTok) over epochs. GQS = Global Quality Scale, JAMA = Journal of the American Medical Association, MAE = mean absolute error, mDISCERN = modified DISCERN.

## 4. Discussion

### 4.1. Overview of main findings

This study systematically evaluated the quality, dissemination ecology, and user feedback of insomnia-related health information on China’s 2 major short video platforms, Bilibili and TikTok. The main findings reveal the intricate relationship between information quality, creator identity, platform characteristics, and user behavior. Overall, the quality of insomnia-related short videos was moderate (median GQS = 3.0; median mDISCERN = 3.5), which is better than assessments for some other disease areas, possibly due to high public attention to insomnia and its relatively mature science communication environment.^[15,16]^ The study reaffirms the core role of source credibility: content created by DHPs significantly outperformed that by nonprofessional creators in terms of information scientificity (mDISCERN) and reliability foundation (JAMA). This conclusion is consistent with findings from short video evaluation studies on topics such as gallstones,^[16]^ diabetes,^[17]^ and attention-deficit/hyperactivity disorder.

The deeper value of this study lies in revealing how platform ecology systematically shapes the presentation, dissemination, and reception of health information. Bilibili and TikTok showed significant differences in video format, interaction patterns, content quality emphasis, and user discussion focus. Crucially, the prediction model based on user comments completely failed (*R*^2^ ≈ 0), strongly suggesting a weak association between online interaction metrics and the objective scientific quality of content, echoing the phenomenon of disconnection between social media engagement and content scientific quality observed in previous studies.^[17,18]^

### 4.2. The shaping of health information dissemination by platform ecology

The observed platform differences can be explained from a health communication theory perspective. Bilibili and TikTok represent 2 distinct logics of health information dissemination. Bilibili users exhibit cognitive information-seeking characteristics, preferring long videos, high rates of collection/commenting, and discussions focused on specific intervention plans (e.g., “Suan Zao Ren Tang”). This aligns with the pursuit of cognitive gratification in the “Uses and Gratifications” theory, serving users’ needs to solve health problems through systematic learning.

In contrast, TikTok serves more to fulfill users’ emotional and social integration needs. Its short videos, high like counts, and comment sections centered on emotional expression reflect motivations for seeking emotional resonance and community belonging. This difference results from the combined effects of platform technological architecture, algorithmic logic, and community culture. TikTok’s algorithm is optimized for capturing instantaneous attention and emotional resonance, shaping a “plaza of emotion” communication scenario.^[19]^ Meanwhile, Bilibili’s community ecology encourages in-depth, structured knowledge sedimentation, constructing an “information repository” environment, as reflected in studies on topics such as COPD.^[20]^

Therefore, platforms are not neutral conduits but structural forces actively defining what constitutes “relevant” and “useful” health information, demonstrating the shaping power of “platform-as-context” on communication practices. Similar content characteristics and platform logic differences have been corroborated in analyses of topics such as inflammatory bowel disease diet^[21]^ and mask promotion.^[22]^

### 4.3. Contradictory findings reveal the complexity of health information dissemination

Several contradictory findings provide a nuanced perspective on the complexity of online health information. First, although professional content is superior in scientificity, videos uploaded by individual users ranked highest in overall perceived quality (GQS) scores.^[15]^ This tension highlights the multidimensionality of evaluation criteria. Individual creators often score high in “attractiveness” and “perceived usefulness” due to excellent storytelling, emotional appeal, and high production quality, but this does not equate to scientific rigor. This suggests that the public and experts may use different criteria for judging “good content,” necessitating a distinction between communication effectiveness and scientific accuracy. Second, the relationship between sharing behavior and information quality showed opposite trends on the 2 platforms, revealing the situational dependence of user sharing motivations. On Bilibili, the number of shares was negatively correlated with video scientificity (mDISCERN), possibly because users share controversial or debate-provoking content to engage in community interaction. On TikTok, shares were positively correlated with information transparency (JAMA), possibly indicating users share videos with clear sources that collectively resonate as a form of emotional expression or social currency.^[17]^ This indicates that “sharing” carries diverse, platform-specific social meanings and should not be simplistically equated with endorsement of scientific value. Finally, the complete failure of the quality prediction model based on user comments points to a core conclusion: in the social media ecosystem, the popularity and emotional resonance of information are often disconnected from its intrinsic scientific quality. User interaction reflects participation and emotional states more than rational quality discernment,^[18]^ a phenomenon also observed in video quality assessments on other topics (e.g., therapies for post-traumatic stress disorder).

### 4.4. Multilevel, differentiated practical implications

Based on the above analysis, targeted and layered practical implications are proposed. First, for platform designers and policy regulators, differentiated governance strategies should be adopted. In knowledge-based communities such as Bilibili, establishing an “Evidence-Based Health” section with algorithmic weighting can incentivize systematic, in-depth science communication content (e.g., CBT-I series courses). On entertainment-oriented platforms such as TikTok, mandatory labels such as “Personal Experience, For Reference Only” should be added to personally experienced health content. Algorithms should also be optimized to ensure fair exposure for “micro-science” content that combines scientific rigor and dissemination appeal from certified professionals. Policy formulation can refer to national guiding principles for all-media health science knowledge dissemination.^[23]^ Second, health professionals and science communicators should master cross-platform narrative skills and implement platform-adapted content strategies: focus on logically rigorous, well-referenced in-depth analysis on Bilibili, while honing emotional, fragmented expression techniques on TikTok, embedding core knowledge into strong narratives or emotional hotspots. All in all, public health institutions and educators should cultivate the public’s “critical platform literacy.” Users should be educated to understand platform-specific information logic differences and learn to cross-verify information credibility based on source credentials (e.g., platform certification, JAMA transparency principles) rather than interaction data. Educational programs can draw on successful social media intervention experiences targeting specific populations.^[24–26]^

### 4.5. Disconnection between user comments, emotional resonance, and scientific quality

The analysis of user comment texts reveals that public interaction with health information exhibits distinct platform-based and emotional characteristics. Bilibili users’ comments focus on specific solutions like “Suan Zao Ren Tang” and “meditation,” reflecting a problem-solving-oriented cognitive information-seeking model. In contrast, TikTok users’ comments are filled with emotional expressions such as “can’t sleep” and “anxiety,” highlighting needs for emotional venting and social support. However, this “heat” or “resonance” based on user expression and interaction behavior shows a profound disconnect from the scientific quality of video content (assessed by experts using GQS, mDISCERN, and JAMA). The prediction model (*R*^2^ ≈ 0) quantitatively confirms this disconnect: regardless of how the model learns from comment features, it cannot effectively predict expert ratings of video scientificity and reliability. This finding is consistent with previous research concluding that on social media, the popularity or interaction metrics of health information often show a disconnect or even negative correlation with its intrinsic scientific quality.^[27,28]^

This disconnection has multiple implications. First, users’ “approval” or “engagement” with health information is primarily based on emotional appeal, narrative attractiveness, or immediate personal relevance (“perceived usefulness”), rather than argumentative rigor, evidence completeness, or source credibility.^[29]^ This reflects that the public is often more easily influenced by surface features and emotional resonance than by in-depth scientific evidence when evaluating online health information.^[30]^ This explains why videos from individual creators (nonprofessionals) can score high on “overall perceived quality” (GQS) but not on “scientificity” (mDISCERN). The public’s “likes,” “favorites,” and empathetic comments essentially reward communication skills and emotional connection strength. This public evaluation system, centered on engagement metrics and emotional responses, coexists with the professional evaluation system based on evidence-based medicine principles emphasizing source reliability and content accuracy, yet their internal logics and value orientations often diverge.^[29,31]^

The disconnection warns that if user interaction data (e.g., comment count, shares) are used as the core or sole weighting factor for algorithmically recommending health information, they may systematically amplify emotionally provocative but scientifically questionable content, exacerbating “infodemic” risks.^[32]^ Therefore, building a healthy online information environment requires not only improving content scientific quality but also introducing a composite evaluation framework in platform mechanism design that balances “emotional resonance” and “scientific value.” Simultaneously, strengthening public education on “critical digital health literacy” is essential to help users distinguish between emotional identification and scientific evidence and cultivate their ability to cross-verify information using multiple criteria.

### 4.6. Research strengths and limitations

The strengths of this study include being the first to conduct a multidimensional comparison of platforms with distinct ecologies on insomnia and innovatively integrating quality assessment with user text analysis. However, several limitations exist: using the single keyword “insomnia” may have missed related videos; the sample is limited to Chinese platforms, affecting conclusion generalizability; although the quality assessment tools (e.g., DISCERN) are widely validated and inter-rater reliability was good (κ ≈ 0.78),^[33]^ scoring remains somewhat subjective; the assessment focused primarily on information content and did not systematically examine narrative structure, visual presentation, or production quality, which also influence communication effectiveness (future research could integrate audiovisual assessment tools); the cross-sectional design prevents causal inference.

Future research can deepen understanding of digital media health communication dynamics through more complex search strategies, larger samples, cross-cultural comparisons, and longitudinal designs. It is worth noting that even short video assessments on topics like thyroid cancer^[34,35]^ face similar methodological challenges, indicating that building a more universal and objective assessment system is a common need in the field.

## 5. Conclusion

This study systematically assessed the quality and dissemination ecology of insomnia-related short videos on TikTok and Bilibili. Core findings indicate that although overall information quality is moderate, platform characteristics, uploader identity, and user behavior collectively constitute a complex information environment. The study confirms that DHPs are key to ensuring content scientificity and reliability, with their content significantly superior to nonprofessional creators in evidence-based grounding.

More critically, this study reveals the powerful shaping force of “platform-as-context”: Bilibili, as an “information repository,” tends to disseminate in-depth, structured science communication content, while TikTok, as a “plaza of emotion,” is optimized for generating broad emotional resonance and instantaneous interaction. A fundamental contradiction emerges – there is a significant disconnect between user interaction metrics (likes, comments, shares) and the scientific quality of content as assessed by experts. This disconnect warns that in the algorithm-driven social media environment, popularity may systematically amplify emotionally provocative but scientifically questionable information, exacerbating “infodemic” risks.

Therefore, building a healthy online information environment requires multilevel, differentiated strategies: platforms need to optimize algorithms to balance “popularity” and “quality” in recommendations; health practitioners need to master cross-platform narrative skills; and enhancing the public’s critical digital health literacy – enabling them to judge information credibility based on source credentials rather than interaction data – has become an urgent public health task. This study provides important empirical evidence for standardizing online health communication and improving public health literacy.

## Author contributions

**Conceptualization:** Yubo Yang, Minghao Zhou, Deyu Jiang, Jingan Tan, Yutao Hou.

**Data curation:** Yubo Yang, Minghao Zhou, Yutao Hou.

**Investigation:** Deyu Jiang.

**Methodology:** Deyu Jiang.

**Project administration:** Yubo Yang, Deyu Jiang.

**Resources:** Minghao Zhou, Deyu Jiang.

**Software:** Minghao Zhou.

**Validation:** Roujia Sha.

**Visualization:** Roujia Sha, Zhiwei Huang.

**Writing – original draft:** Yubo Yang, Zhiwei Huang, Jingan Tan, Yutao Hou.

**Writing – review & editing:** Yubo Yang, Deyu Jiang, Roujia Sha, Zhiwei Huang, Jingan Tan, Yutao Hou.


